# Signaling as a context-dependent strategy in action control

**DOI:** 10.1007/s00426-025-02145-w

**Published:** 2025-06-23

**Authors:** Lorena Hell, Christoph Felix Geissler, Philip Schmalbrock, Christian Frings

**Affiliations:** 1 Department of Cognitive Psychology, Trier University, Universitätsring 15, Building D, 54296 Trier, Germany; 2 Institute for Cognitive and Affective Neuroscience (ICAN), Trier University, Universitätsring 15, Building D, 54296 Trier, Germany

## Abstract

In sequential two-choice tasks, performance is typically improved when both the stimulus features and the response are repeated but worsens when only one of them repeats and the other changes (partial repetition costs). The signaling account assumes a bias of response selection towards a repetition or change by applying a heuristic that uses the relation between previous and current stimulus features as a response signal. We investigated whether the signaling heuristic is modulated by contextual information, specifically the comparability of different display set sizes that signal a response with either few or many stimuli. Participants worked through a sequential task while display set sizes were varied within (Exp. 1, *N* = 45; enabling comparison between displays), or between participants (Exp. 2; *N* = 130, enabling no comparison between displays). Contrary to findings in typical two-choice tasks, partial repetition costs were not observed with small set sizes and only emerged at larger set sizes in Experiment 1 but were similar in Experiment 2. These results suggest that signaling incorporates context information to adapt the usage of stimulus information for response strategies in accordance with the environment.

## Introduction

In everyday life, individuals face a range of objects that provide different action possibilities (Gibson, 1977). Yet, even actions as simple as pressing a key, although appearing trivial, involve intricate action control mechanisms (Frings et al., 2024) that are crucial for effective and goal-directed interaction with the environment. One widely researched aspect is the influence of past stimulus-action episodes on the current action. It is assumed that features of stimuli and motor actions are stored in a common representation, which can be retrieved in a subsequent episode and influences the outcome of the action (Frings et al., 2020; Hommel, 1998, 2004, 2019; Prinz, 1997). Another relevant aspect is the coding of abstract relational codes, such as whether stimuli or responses from previous episodes are perceived as repeating or changing (Hazeltine et al., 2024).

Stimulus-action sequences are often investigated in so-called partial repetition tasks, such as the S1R1-S2R2 task (Hommel, 1998) or the distractor-response binding task (DRB; Frings et al., 2007). For instance, in the DRB task, a target stimulus has to be identified and responded to. In addition, one or several to-be-ignored distractors accompany the target. Importantly, the response and distractor relation are varied between subsequent prime and probe displays in a single trial, resulting in four possible conditions where either target and distractor can both repeat, both change, or only one of them repeats. Typically, it is observed that a partial repetition between prime and probe leads to a significant decrease in performance compared to the other conditions. These performance costs are referred to as partial repetition costs (PRC, Hommel, 1998). Two mechanisms have been proposed as a reason for these partial repetition costs: unbinding (Hommel, 2004) and signaling (also described as a ‘bypass rule’; Fletcher & Rabbitt, 1978; Frings et al., 2007; Weissman et al., 2023), and both mechanisms seem to contribute to this effect (Weissman et al., 2023).

The basic assumption of the *(un-)binding hypothesis* is that the target, motor action, distractor(s), and potentially other context information are bound together in a common short-term memory trace called event file, which is later retrieved and interacts with the current action episode (Beste et al., 2023; Frings et al., 2020; Hommel et al., 2001; Hommel, 2004; but see Mocke et al., 2023). In case of a full repetition, the retrieved event file improves performance because its features match the current action episode. However, in case of a partial repetition, the event file negatively interferes with the current action episode, because a part of the current display is still representationally connected to a previous response that is inappropriate in the current episode. In order to overcome this interference, the link established in the prior episode first must be ‘unbound’. Accordingly, it has been suggested that one stimulus can only be involved in one binding at a time and has to be unbound before it can be used to form a new binding in the current action episode again (Hommel, 2004).

The *signaling hypothesis* offers a different perspective on partial repetition costs (Fletcher & Rabbitt, 1978; Weissman et al., 2023), in the past also referred to as the bypass rule (Frings et al., 2007). This hypothesis proposes that participants apply a simple decision heuristic, namely whether the ‘signal’ (i.e., the stimuli) repeats or changes between trials and bias their response accordingly (Fletcher & Rabbit, 1978; Jones et al., 2013; Williams, 1966). In many PRC tasks, this strategy allows to bypass the identification process in favor of a simple rule (if stimuli repeat, press the same response as before; if they change, give the other response). This rule is adaptive, particularly in tasks with only two response choices, since a stimulus change implies a specified response alternation.

In summary, partial repetition tasks—of which DRB is one example—typically result in partial repetition costs. These costs can be attributed to either binding processes (e.g., Frings et al., 2007) or signaling processes (e.g., Weissman et al., 2023). In general, both mechanisms can contribute to the emergence of partial repetition costs (Weissman et al., 2023). In this work, we focus on the signaling process and how the use of such a heuristic is influenced by context and experience within the experimental setup. Specifically, we are interested in how context shapes the experience and the (implicit or explicit) knowledge about the nature of episode sequences, which then influences the likelihood of applying the signaling heuristic.

### Context influences on the signaling hypothesis

To the best of our knowledge, only a few studies have been published that investigate this specific aspect in signaling heuristics. Fletcher and Rabbitt (1978) explored practice (in terms of more experience and time spent with the task) as a factor that influenced selection biases for comparison strategies. In their study, participants completed a forced-choice task with two response alternatives in sequences of letter stimuli. Sporadically, another noisy dot pattern stimulus appeared, and participants were given a free response choice between the two prior alternatives. At the beginning of the experiment, unpracticed participants tended to repeat their response from the previous trial, whereas practiced participants treated the noise stimuli as a change and pressed a different button. The authors concluded that response strategies were formed by participants and changed over time with practice, until a change or repetition in stimulus material was reflected in the participants’ response selection.

According to Todkill and Humphreys (1994), the nature of the specific comparison strategy is subject to factors in the experimental context, such as the rate of the stimulus presentation. At rapid rates, the typical previously described bypass strategy is used to compare a currently present to an immediately prior presented stimulus. At slow rates in contrast, a more long-termed strategy is applied that compares the current stimulus to a template stored in long-term memory (Todkill & Humphreys, 1994).

Emphasizing the importance of context and experience with the experimental setup, Jones et al. (2013) showed in a perceptual categorization task that performance in sequential tasks can be explained by two mechanisms that reflect the participant’s learning of statistics of response and stimulus rates: learning the base rate (i.e., in how many trials a stimulus or response occurs) and the repetition rate (i.e., how often displays repeat compared to the previous one). They also identified stimulus discriminability as an experimental factor that influences the participant’s expectancy of the repetition rate.

### The present study

In summary, partial repetition tasks—of which DRB is one example—typically lead to partial repetition costs (Fletcher & Rabbitt, 1978; Frings et al., 2007). These costs can arise from binding processes (Frings et al., 2007) or signaling processes (Weissman et al., 2023), and both mechanisms may contribute to their occurrence (Weissman et al., 2023). However, this study specifically focuses on the signaling process and its modulation by context and experience within the experimental setup.

To emphasize signaling while minimizing the influence of binding, we designed a task that strongly encourages a signaling strategy. This was achieved by implementing an easy discrimination task with only two response choices, which facilitates the use of signaling heuristics. The effects of signaling manifest as partial repetition costs, as well as complete change and complete repetition benefits. Prior research has shown that tasks with four response choices—a typical feature in distractor-response-binding studies—reduce the impact of signaling (Hazeltine et al., 2024). Additionally, we apply a sequential task structure with commonly used partial repetition tasks in signaling research, which have been shown to reliably produce partial repetition costs attributed to signaling strategies (Weissman et al., 2023).

Research suggests that the experimental context can shape how signaling heuristics are applied in sequential tasks. In our study, we investigate whether the experimental context influences the extent to which participants rely on signaling heuristics in a partial repetition task. Specifically, we examine how the perceived similarity between consecutive displays—modulated by the experimental context—affects heuristic-based response biases.

**Fig. 1:**
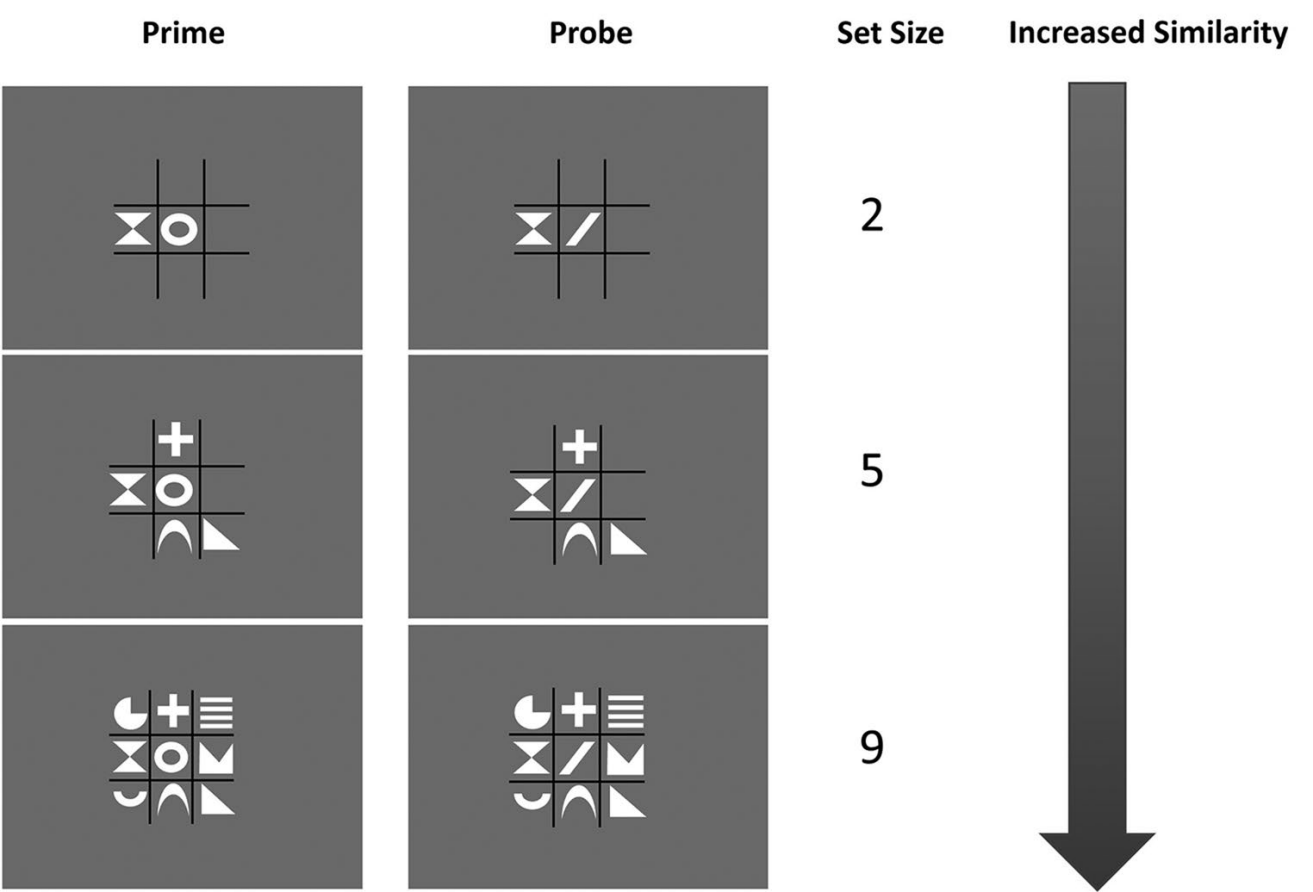
Set Size Conditions and Similarity Note. Comparing repetitions between prime and probe, perceived similarity increases with larger set sizes

Since participants use a signal to guide their responses, greater similarity between two displays increases the likelihood that the signal is perceived as repeating, leading to a higher tendency to repeat the response as well. To manipulate perceived similarity, we systematically varied distractor set size (see Fig. 1). This is based on the assumption that a larger proportion of distractor repetition (or change) enhances the overall similarity of consecutive displays. For instance, in a small set size (one target, one distractor), a partial repetition results in exactly 50% of the display repeating. However, in larger set sizes, the weight of distractors on the response signal increases—meaning a greater portion of the display repeats or changes. In the largest set size, up to 89% of the display may repeat or change, strengthening the signal and increasing perceived similarity. As a result, we predict that partial repetition costs will be larger in large set sizes compared to small ones.

Importantly, we assume that the set size effect is moderated by the experimental context, particularly by the comparison between set sizes within participants. When both small and large set sizes are presented within the same experiment, their comparison influences how similarity is perceived. In this context, a display with two repeating stimuli may be perceived as less similar when participants also encounter displays where eight stimuli repeat. This difference in perceived similarity and the resulting partial repetition costs should not emerge in a context where participants are only exposed to a single set size, as they lack a basis for comparison between different display sizes.

To test these hypotheses, we varied the number of distractor stimuli that were repeated from prime to probe in a partial repetition task (DRB, Frings et al., 2007). The signaling hypothesis predicts larger PRC in these circumstances. In the context of the literature it seems important whether participants perceive displays with different set sizes of stimuli as more similar or not. Thus, we varied the number of repeating stimuli within and between subjects. If the experimental context influences the perception of similarity and hence induces a signaling heuristic, a display with 2 repeating stimuli should be perceived as less similar in a context in which displays with 8 repeating stimuli are present as well.

As a partial repetition task, we employed the DRB task (Frings et al., 2007) that involves a sequential design featuring a prime display followed by a probe display. Each display contains both a target stimulus and irrelevant distractor stimuli. Participants are required to identify the target by pressing a button, while ignoring the distractors. The relationship between the response to the target and the distractors from the prime to the probe is varied orthogonally: both the response (either repeating (response repetition, RR) or changing (response change, RC) and the distractors (either repeating (distractor repetition, DR) or changing (distractor change, DC) can differ. The application of signaling heuristics results in a response bias: a tendency to change responses when a feature in the display changes and to repeat responses when all features remain the same. Consequently, partial repetitions (RRDR or RCDR) incur performance costs compared to full repetition or full change conditions (RRDC or RCDC; e.g., Weissman et al., 2023). To comprehensively capture this effect, we introduced the *Partial Repetition Cost Index (PRC index)*, which quantifies the aggregated difference between costs and benefits. This index is computed as (RRDC – RRDR) – (RCDC – RCDR), following the standard approach of calculating costs and benefits in the DRB paradigm (Frings et al., 2007).

According to our hypothesis, the experimental context influences how the signaling heuristic is applied to different set sizes in the sense that the contribution of the signaling strategy in one set size depends on a comparison to other set sizes if this information is available. In a within-subjects design with an exposure to different set sizes (Experiment 1), a large set size with many simultaneous repeating or changing distractors has a strong affordance for signaling and has a higher perceived similarity compared to a smaller set size, which should lead to larger PRC for the larger set size. To the contrary, in a between-subjects design where only a single set size is presented to a participant and such a set size comparison cannot occur (Experiment 2), a similar contribution of signaling is assumed for all set sizes that should result in similar PRC.

To foreshadow the results, PRC in Experiment 1 increased as a function of set size. With a set size of 2 stimuli in this experimental context, no PRC occurred while for a set size with 8 stimuli PRC occurred (with set size 5 falling in between). Yet, in Experiment 2, where set size was varied between participants, DRB effects for set size 2 versus 8 did not differ.

## Experiment 1

### Method

#### Participants

45 participants were recruited from the platform Prolific (www.prolific.com) and participated online. Data of four participants were excluded due to being a heavy outlier regarding reaction times following the taxonomy by Tukey (1977). Specifically, we excluded participants whose reaction times or error rates exceeded three times the interquartile range from the median of the entire sample. This resulted in a final set size of *n* = 41. The median age was 25 years (mean: 29.1, range: 20–57). 20 participants were female, and 35 participants reported right-handedness. South African was the most reported nationality (13 subjects), followed by Portuguese (12 subjects) and Polish (5 subjects). All participants reported normal or corrected-to-normal vision, fluency in the English language and no dyslexia, past head injury, cognitive impairment, ADD/ADHD, or any mental health diagnosis. Participants were compensated with £6.75 for 45 min under the condition that they made less than 20% of errors throughout the experiment (since the task is very simple, a high error rate indicates that data include random responses). The study was conducted according to ethical guidelines of Trier University. Participants consented to participate and to the use of their data for scientific purposes in this study. The data were gathered in May 2023. The sample size calculation was based on previous studies investigating the DRB effect as an analogous measure of capturing performance costs and benefits, which typically led to medium-sized effects (*d* = 0.50). Thus, we planned to run at least 38 participants, leading to a power of 1 − β = 0.85 (assuming an α = 0.05; G*Power 3.1.9.2; Faul et al., 2009). Additional participants were recruited due to anticipated dropouts.

#### Design

The 3 × 2 × 2 experimental design comprised the following within-subjects factors: set size (2 vs. 5 vs. 9), response relation (response repetition vs. response change from prime to probe), and distractor relation (distractor repetition vs. distractor change from prime to probe).

#### Materials

The experiment was programmed using PsychoPy (Peirce et al., 2019; version 2022.2.5) and run online via Pavlovia (Peirce & MacAskill, 2018). PC or notebook were allowed for participation. Instructions and stimuli were shown in white on grey background. 18 white (RGB_255_: 255, 255, 255) shapes (e.g., triangle, circle, star) were used in total with up to 9 shapes appearing in one single display. Each shape had the size of 25 × 25 px. The shapes were separated by a black (RGB_255_: 0, 0, 0) grid. The size of the complete display measured 100 × 100 px. Participants responded by pressing one of two keys (F or J) on the computer keyboard with their index fingers.

#### Procedure

Participants registered for the study and received the study link from the recruitment platform used at Trier University (Sona Systems; www.sona-systems.com). Participants first indicated their sex, age, handedness, and native language before the start of the experiment. Instructions were given on screen. Participants were instructed to place their left index finger on the F key and the right index finger on the J key. Their task was to categorize two shapes by pressing the corresponding key (F or J). For each participant, two of 18 available shapes were randomly selected as their individual target shapes with the rest of the shapes serving as distractor material.

**Fig. 2:**
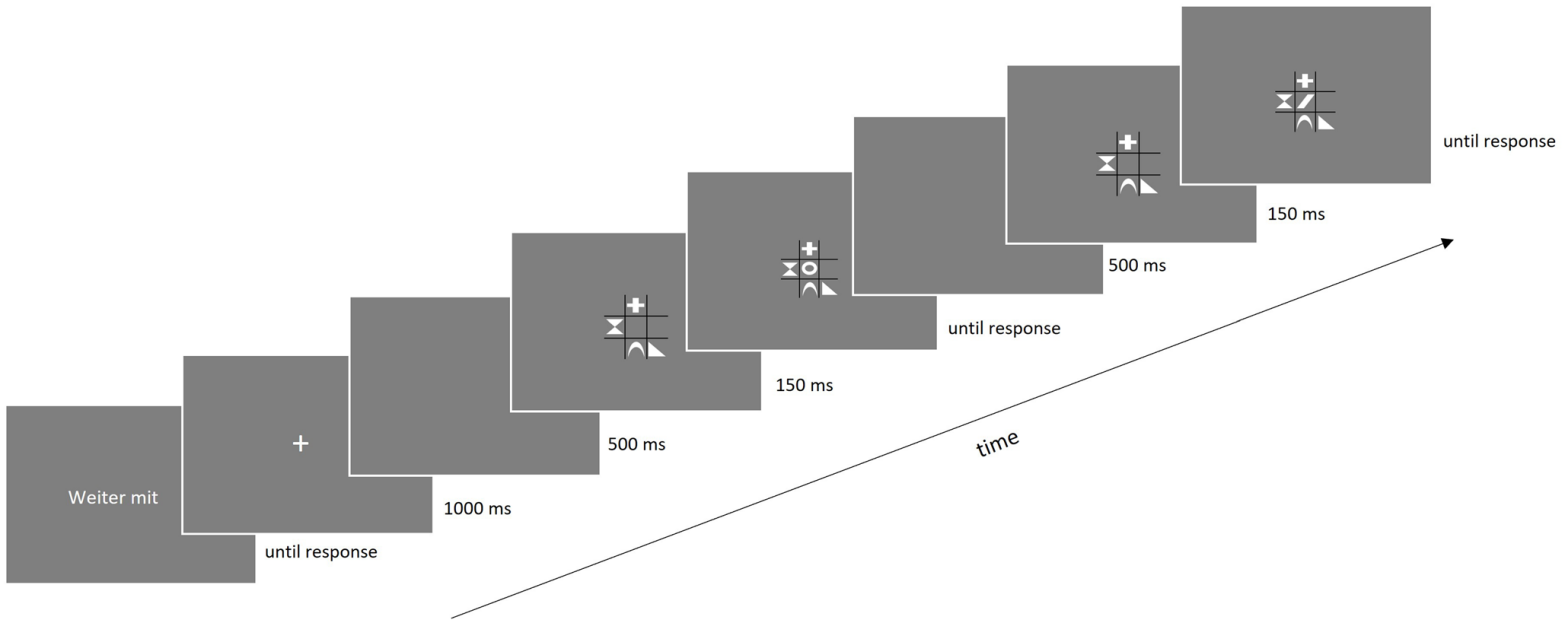
Exemplary Experimental Trial Note. This trial depicts a response change and distractor change condition for all three set sizes

Each trial consisted of two consecutive responses: in both the prime and the probe, the identity of the target had to be categorized, which was centrally located next to one or several distractor shapes in a black grid. One trial comprised the following sequence of events (see Fig. 2): Participants initiated each trial manually by pressing the space bar. A fixation mark appeared for 1000 ms, followed by a blank interval of 500 ms. The prime display contained the centrally presented target shape and one, four or eight distractor shapes. Prime distractors were randomly chosen from the 16 non-target shapes and were displayed at random (not central) positions in the grid. With a delay of 150 ms, the central target shape was then simultaneously presented alongside the distractors until a response was made, requiring a correct key press according to the target’s identity (F or J). After an interstimulus interval of 500 ms, the probe display appeared with one, four or eight distractors in a grid, followed by a central target shape after 150 ms which was presented simultaneously with the distractors until response. In the response repetition (RR) condition, the probe target shape was the same as in the prime, and in the response change (RC) condition, the probe target was another of two possible target shapes. In the distractor repetition (DR) condition, all distractors were repeated from prime to probe, and in the distractor change (DC) condition, all distractor shapes changed. The set size as well as the positions of the target and distractors remained throughout the same trial, but randomly varied between trials.

The set size as well as the response relation and distractor relation conditions were varied on a trial-by-trial basis within-participants. Each set size included an equal number of condition combinations for both the response relation and distractor relation conditions. Participants completed 10 practice trials randomly chosen from all conditions before the start of the experiment. Feedback was applied for correct and wrong responses during practice. In the experiment, feedback was only given in case of an incorrect response. Participants had opportunities to pause between trials due to the self-paced trial start. Each subject completed 384 trials in total. Overall, there were 32 repetitions per condition. The whole experiment lasted about 45 minutes.

#### Data processing

Data were processed and analyzed using R (R Core Team, 2022; version 2023.03.2). The package ‘dplyr’ (Wickham et al., 2019) served for data processing and aggregation, and the package ‘ez’ (Lawrence, 2016) was used to compare experimental conditions via a repeated-measures analysis of variance (ANOVA) with type III sum of squares. Regarding RTs, trials with either a wrong prime or probe response were discarded (3.97%). For ERs, trials with incorrect prime responses were not considered (2.28%). Further, reaction times below 200 ms and above 1.5 interquartile ranges over the third quartile of each person’s RT distribution were excluded for the analyses (Tukey, 1977), 5.41% in total. In sum, 9.38% of all trials were excluded. The Partial Repetition Cost index (PRC index) was calculated for RTs and ERs using the following formula: (RRDC – RRDR) – (RCDC – RCDR).

### Results

#### Reaction times

Probe RTs were analyzed in a 3 (set size: 2 vs. 5 vs. 9) × 2 (response relation: repetition vs. change) × 2 (distractor relation: repetition vs. change ) repeated-measures ANOVA. There was a significant main effect of response relation, *F*(1, 40) = 47.23, *p* <.001, η_p_² = 0.54, indicating faster responses in case of repetitions (433 ms) compared to changes (471 ms), and a main effect of set size, F(2, 80) = 6.34, *p* =.003, η_p_² = 0.14, with faster responses for the large set size (450 ms) than for the lower (455 ms) and middle set size (452 ms). The main effect of distractor relation did not reach significance, *F*(1, 40) = 2.20, *p* =.146, η_p_² = 0.05, evidencing response times did not significantly differ depending on repetition or change. The interaction of response relation and distractor relation was significant, *F*(1, 40) = 14.05, *p* <.001, η_p_² = 0.26, suggesting overall effects due to partial repetitions. The interaction set size × response relation × distractor relation was significant, *F*(2, 80) = 3.27, *p* =.043, η_p_² = 0.08, suggesting that PRC were modulated by set size.

**Fig. 3:**
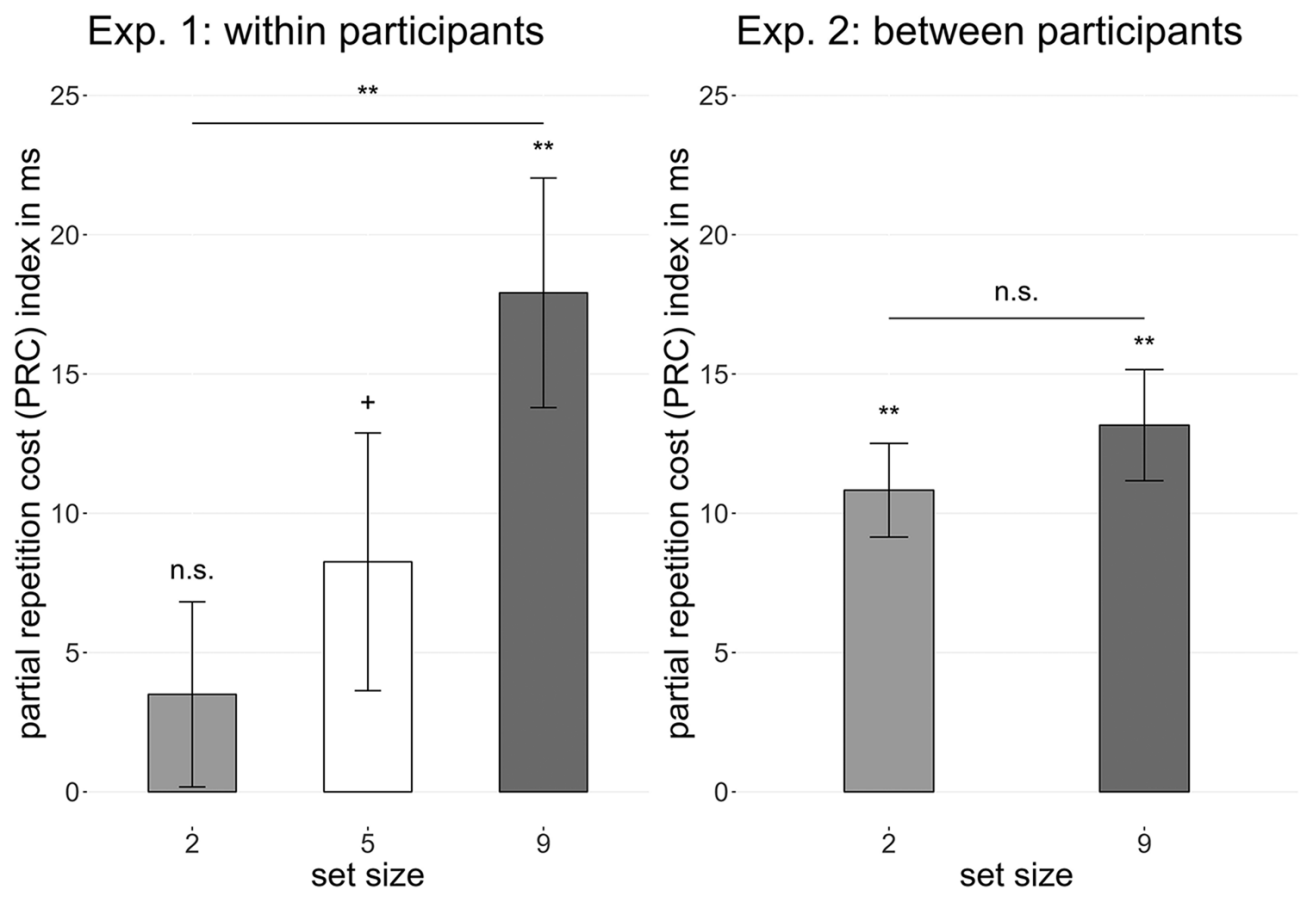
Mean PRC indices for Experiment 1 (within participants) and 2 (between participants) as a function of set size Note. Error bars depict within-participants standard error of the mean (Morey, 2008)

For the three comparing tests of the PRC index between the set sizes, the Bonferroni correction resulted in an adjusted α = 0.017. Paired *t* tests revealed no significant difference in the PRC index between set size 2 and 5, *t*(40) = 0.84, *p* =.405, *d* = 0.13, nor between set size 5 and 9, *t*(40) = 1.46, *p* =.152, *d* = 0.23. However, the difference between set size 2 and 9 was significant, *t*(40) = 0.3.00, *p* =.005, *d* = 0.47 (see Fig. 3). For each set size, the PRC index was computed and tested in a one-sample *t* test against 0. The PRC index for set size 2 was not significant, *t*(40) = 0.852, *p* =.399, *d* = 0.13, and set size 5 reached marginal significance, *t*(40) = 0.1.72, *p* =.092, *d* = 0.27, whereas the PRC index for set size 9 was significant, *t*(40) = 4.79, *p* <.001, *d* = 0.75.

#### Error rates

Regarding the repeated-measures ANOVA, the main effect of response relation was significant, *F*(1, 40) = 8.59, *p* =.006, η_p_² = 0.18), with more errors occurring in response changes (2.14%) than in response repetitions (1.35%). The main effects for distractor relation, *F*(1, 40) = 2.11, *p* =.154, η_p_² = 0.05, and set size, *F*(2, 80) = 2.19, *p* =.119, η_p_² = 0.05) did not reach significance, indicating that that the repetition or change of distractors had no influence on probe error rates (repetition: 1.60%, change: 1.88%), and probe errors did not vary across set sizes (set size 2: 1.81%, set size 5: 1.45%, set size 9: 1.96%). The interaction of response relation and distractor relation was marginally significant, *F*(1, 40) = 3.68, *p* =.062, η_p_² = 0.08, showing that when both the response and distractor repeated, less errors were made than when only one of them repeated. The interaction between set size, response relation and distractor relation did not yield significance, *F*(2, 80) = 1.74, *p* =.181, η_p_² = 0.04, indicating that partial repetition costs were not modulated by the set size of the display.

Paired *t* tests showed that the PRC index of set size 2 did not significantly differ from set size 5, *t*(40) = 0.27, *p* =.786, *d* = 0.04. The difference between set size 2 and 9 is also not significant, *t*(40) = 1.58, *p* =.122, *d* = 0.25, and the middle set size of 5 and large set size of 9 did not significantly differ as well, *t*(40) = 0.1.77, *p* =.085, *d* = 0.28. The PRC index was tested for each set size with student’s one-sample *t* tests. The PRC index was neither significant for set size 2, *t*(49) = 0.43, *p* =.668, *d* = 0.07, nor for the middle set size of 5, *t*(40) = 0.01, *p* =.995, *d < 0.01*), but was significant for the large set size of 9, *t*(40) = 2.63, *p* =.012, *d = 0.41*). For the three comparing tests of binding effects between the set sizes, the Bonferroni correction resulted in an adjusted α = 0.017.

**Fig. 4:**
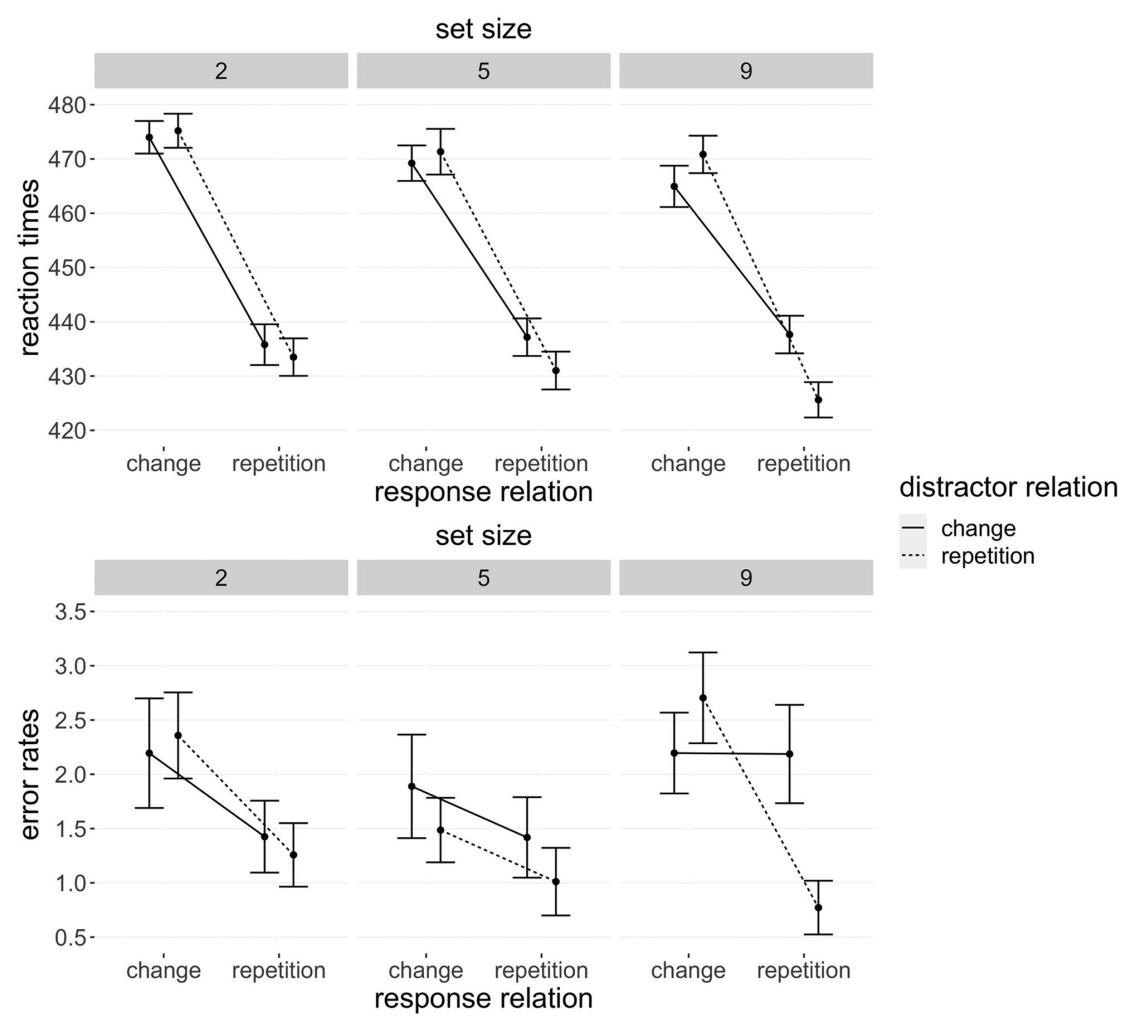
Mean values of both dependent variables for every condition in Experiment 1 Note. Error bars depict within-participants standard error of the mean (Morey, 2008)

Further details on reaction times and error rates with means for response and distractor relations are shown in Fig. 4.

## Discussion

Experiment 1 investigated the effect of experimental context on signaling heuristics in a DRB task with set size as a within-participants factor. We used the PRC index with a maximized affordance for a strategic signaling approach as an index for differences between partial repetition costs compared to full repetition benefits and full change performance. The PRC index was not significant at the low set size of 2 and only emerged at higher set sizes in a staircase-like pattern, with the largest set size of 9 revealing the strongest effect.

At first glance, it seems uncommon that the low set size of 2 did not result in partial repetition costs or full repetition benefits that are sufficient to produce a PRC index, because this set size condition is, of all set sizes, the most comparable to typical partial repetition and DRB tasks, which typically produce reliable costs and benefits using only few stimuli (e.g., Frings et al., 2007, Schmalbrock et al., 2023). One important distinction between Experiment 1 and other typical DRB studies which demonstrate partial repetition costs with a set size of two is that, in the other studies, participants were able to anticipate the set size.

However, the same condition with same stimuli revealed significant DRB effects (computed analogously to our PRC index) in another DRB study in the context of a different hypothesis (Hell et al., submitted); in that study, set size could also not be anticipated. Crucially, that study did not maximize the affordance for a signaling heuristic, because distractors never all repeated and thus, a signaling strategy did not have beneficial value for the task. This implies that the diminishing partial repetition costs are not solely attributable to set size anticipation. Rather, it seems to be influenced more substantially by the potential to use a signaling heuristic, which arises from the high degree of display similarity when set sizes are directly comparable. Therefore, we emphasize that the given result pattern of Experiment 1 does not fit the predictions of the binding hypothesis and rather mainly reflects performance outcomes due to signaling heuristics.

The data therefore indicate that, in the case of high signaling affordance, not only the current episode and condition determine the use of a bypass rule. Rather, the current action episode (and set size) is compared to previously exposed conditions (other set sizes) and the influence of a signaling strategy on performance is tuned to each condition based on the overall comparison. In other words, participants seem to use the number of repeating stimuli as a factor in signaling heuristic. In this way, sequential effects in partial repetition tasks become smaller or diminished for a small set size when compared to larger set sizes in a within-subjects design. The inclusion of larger set sizes within the experimental context appears to redefine the reference point for what qualifies as a useful ‘signal’ in heuristic decision-making. In other words, when participants can compare large set sizes, their heuristic strategy adapts to require a greater amount of information to influence responses. Consequently, this shift reduces or eliminates the application of the heuristic to smaller set sizes.

To test this claim, we repeated the experiment using a between-participants design for set size instead of the previously used within-participants design. We only incorporated set size 2 and 9 in Experiment 2 due to their significant difference in Experiment 1. According to our hypothesis, we expect no difference in the PRC index to occur between both set sizes, because participants have no opportunity for a set size comparison and thus, the signaling process will only adapt to the one presented set size condition, resulting in similar effects for both conditions.

## Experiment 2

### Method

#### Participants

130 participants were recruited from the platform Prolific (www.prolific.com) and participated online. Data of four participants were excluded due to being a heavy outlier regarding reaction times or error rates following the taxonomy by Tukey (1977). Specifically, we excluded participants whose reaction times or error rates exceeded three times the interquartile range from the median of the entire sample, resulting in a final set size of *n* = 126. The median age was 29 years (mean: 32.1, range: 19–72). 62 participants were female, 63 male and 1 of other sex. 114 participants reported right-handedness and 2 reported to be both-handed. South Africa was the most reported nationality (45), followed by Poland (12), United Kingdom (11), Greece (10) and Italy (8). All participants reported normal or corrected-to-normal vision, fluency in the English language and no dyslexia, past head injury, cognitive impairment, ADD/ADHD, or any mental health diagnosis. Participants were compensated with £6.75 for 45 min under the condition that they made less than 20% of errors throughout the experiment (since the task is very simple, a high error rate indicates that data include random responses). The study was conducted according to ethical guidelines of Trier University. Participants consented to participate and to the use of their data for scientific purposes in this study. The data were gathered in July 2023. The sample size was computed according to the medium-sized effect (*d* = 0.45) found for the difference between the small and large set size in RT in Experiment 1. Thus, we planned to run at least 124 participants, leading to a power of 1 − β = 0.80 (assuming an α = 0.05; G*Power 3.1.9.2; Faul et al., 2007). Additional participants were recruited due to anticipated dropouts.

#### Design

The design was identical to the design of Experiment 1 with the exception that the set size only contained 2 levels (small set size 2 vs. large set size 9) and was applied between participants. This resulted in a 2 × 2 × 2 design with the factors: set size (2 vs. 9; between-participants), response relation (response repetition vs. response change from prime to probe; within-participants), and distractor relation (distractor repetition vs. distractor change from prime to probe; within-participants).

#### Materials

The materials were identical to the materials of Experiment 1.

#### Procedure

The procedure was identical to that used in Experiment 1 except that participants were randomly assigned to only one single set size condition for the entire experiment.

#### Data processing

The data processing procedure was identical to the procedure of Experiment 1. Regarding RTs, trials with either a wrong prime or probe response were discarded (3.05%). For ERs, trials with incorrect prime responses were not considered (1.74%). Further, reaction times below 200 ms and above 1.5 interquartile ranges over the third quartile of each person’s RT distribution were excluded for the analyses (Tukey, 1977), 4.51% in total. In sum, 7.56% of trials were discarded.

### Results

#### Reaction times

Probe RTs were analyzed in a 2 (set size: 2 vs. 9) × 2 (response relation: repetition vs. change) × 2 (distractor relation: repetition vs. change) mixed-measures ANOVA. There was a significant main effect of response relation, *F*(1, 124) = 146.96, *p* <.001, η_p_² = 0.54, indicating faster responses in case of repetitions (461 ms) compared to changes (510 ms). Distractor relation did not reach significance, *F*(1, 124) = 1.51, *p* =.221, η_p_² = 0.01, evidencing response times did not significantly differ depending on repetition (485 ms) or change (486 ms) of the distractor. The main effect of set size was also not significant, F(1, 124) = 0.01, *p* =.924, η_p_² < 0.01, indicating no difference between response times for the small (485 ms) and the large (486 ms) set size. The interaction of response relation and distractor relation was significant, *F*(1, 124) = 40.86, *p* <.001, η_p_² = 0.25, suggesting overall partial repetition costs. The interaction set size × response relation × distractor relation was not significant, *F*(1, 124) = 0.39, *p* =.535, η_p_² < 0.01, evidencing that the PRC index was not moderated by set size.

A *t* test for independent samples revealed no significant difference in the PRC index between set size 2 and 9, *t*(121.73) = 0.62, *p* =.539, *d* = 0.06. For each set size, the PRC index was computed and tested in a one-sample *t* test against 0. The PRC index for set size 2 was highly significant, *t*(61) = 4.44, *p* <.001, *d* = 0.56, as well as the PRC index for set size 9, *t*(63) = 4.63, *p* <.001, *d* = 0.58.

#### Error rates

For the analysis of probe error rates, a similar pattern regarding the PRC index and set size emerged. Regarding the ANOVA, there was a significant main effect of response relation, *F*(1, 124) = 44.84, *p* <.001, η_p_² = 0.27, indicating a larger number of errors in the response change condition (1.93%) compared to the response repetition condition (0.76%). Distractor relation did not reach significance, *F*(1, 124) = 2.66, *p* =.105, η_p_² = 0.02, evidencing error rates did not significantly differ depending on repetition (1.46%) or change (1.23%) of the distractor. The main effect of set size was also not significant, F(1, 124) = 1.97, *p* =.163, η_p_² = 0.02, indicating no difference between error rates for the small (1.51%) and the large (1.19%) set size. The interaction of response relation and distractor relation was significant, *F*(1, 124) = 32.91, *p* <.001, η_p_² = 0.09, suggesting overall partial repetition costs. The interaction set size × response relation × distractor relation was not significant, *F*(1, 124) = 0.11, *p* =.741, η_p_² < 0.01, evidencing that the PRC index was not moderated by set size.

A *t* test for independent samples revealed no significant difference in the PRC index between set size 2 and 9, *t*(114.21) = 0.33, *p* =.742, *d* = 0.03. The PRC index was tested for each set size with student’s one-sample *t* tests. The PRC index for set size 2 was significant, *t*(61) = 2.41, *p* =.019, *d* = 0.31, as well as the PRC index for set size 9, *t*(63) = 2.65, *p* =.010, *d* = 0.33.

**Fig. 5:**
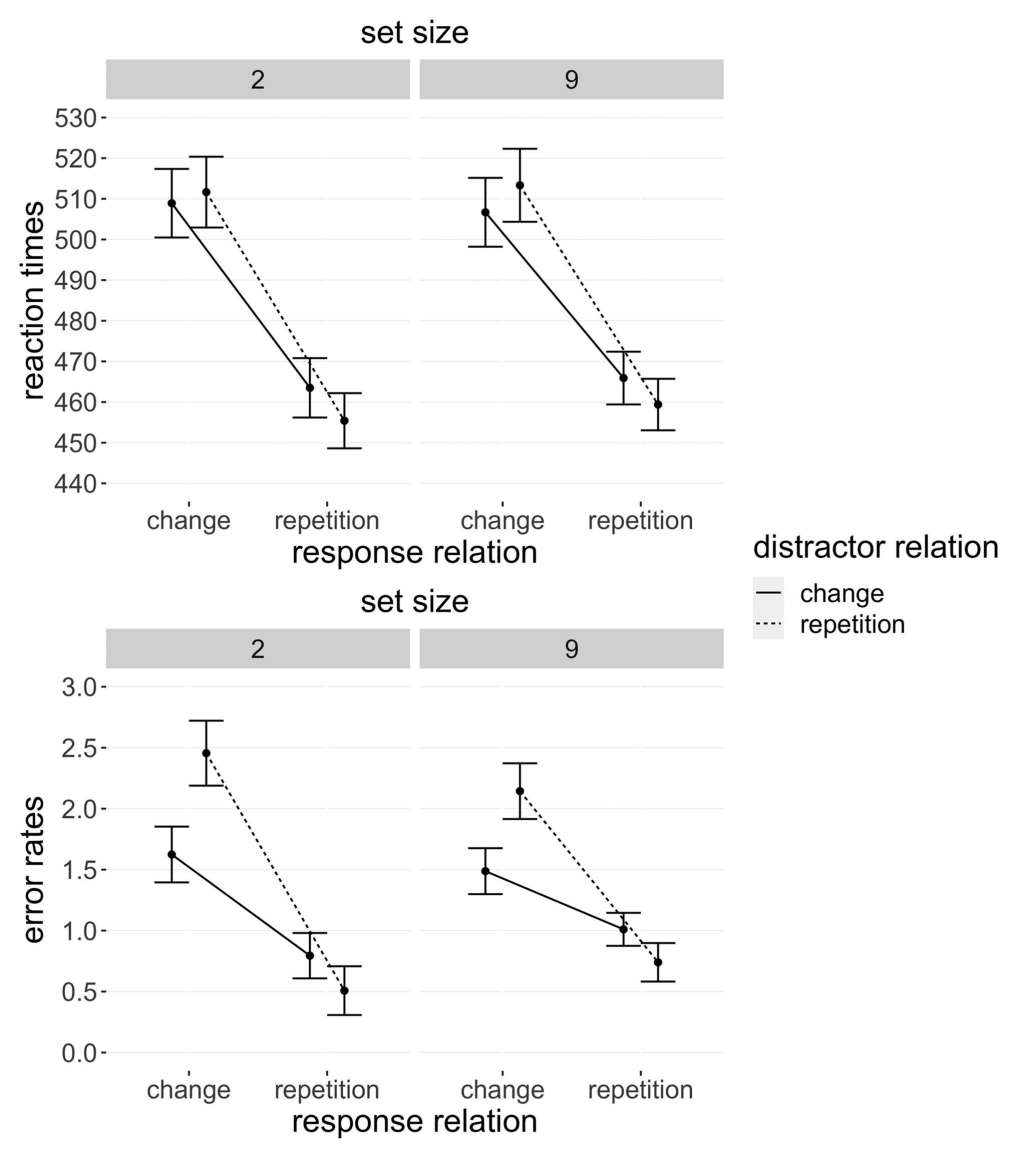
Mean values of both dependent variables for every condition in Experiment 2 Note. Error bars depict within-participants standard error of the mean (Morey, 2008)

Details of reaction times and error rates with response and distractor averages are shown in Fig. 5.

### Discussion

Experiment 2 replicated the study design of Experiment 1 with the important change of applying a between-participants instead of the within-participants design. Partial repetitions costs in form of the PRC index emerged at the low set size of 2 (in contrast to their absence in the within-participants design of Exp. 1) and, furthermore, were not significantly different from the large set size of 9. It is important to emphasize that all set size conditions were identical in both experiments and thus, the appearance of partial repetition costs at a low set size is attributed to the switch from a within- to a between-participants design.

In line with our hypothesis, the size of the PRC index was affected by the experimental context. The observed pattern is not explainable with a pure binding approach, because the binding hypothesis does not predict a larger index of costs and benefits (DRB effect) with larger set sizes; to the contrary, DRB effects in binding studies are even reduced with larger set sizes due to a limitation in the integration or retrieval of distractors (Hell et al., submitted; Schmalbrock et al., 2023).

However, the signaling hypothesis predicts higher partial repetition costs in such a condition, when different set sizes can be compared to each other over the course of an experiment. If participants depend on a ‘signal’ to influence their response, a greater similarity between two consecutive displays results in a higher chance that the signal is perceived as repeating (or changing), which in turn biases the response to be repeated (or changed) as well. In this specific case, the greater similarity is achieved by a larger amount of stimulus information (with larger set sizes), which leads to a more pronounced influence of the signaling heuristic compared to the smaller set size when both set sizes are compared. The fact that the PRC index in the small set size differs between experiments suggests that the within-subjects design leads to ‘tuning’ of the signaling strategy to different set sizes as a result of a comparison process.

### General discussion

We investigated how different experimental contexts lead to different applications of signaling strategies in a partial repetition cost/DRB task. Two experiments revealed significantly different patterns in the PRC index for identical conditions, depending on whether they are embedded in a within-participants or between-participants design. First, the distractor set size was manipulated within-participants to enable a comparison process, which would in turn lead to a tuning of the signaling heuristic. This process involved a higher similarity between prime and probe displays for the large set size (with more distractor information indicating repetitions or changes) resulting in a higher likelihood of a response bias towards repetition or change.

Indeed, the PRC index increased with set size in a within-participants design, showing a nonsignificant PRC index in the smallest set size and a significant increase with larger set sizes. Secondly, in contrast to the findings in the within-participants design, these differences in the PRC index were not observed in a between-participants design, in which the small and large set size resulted both in a significant and similar PRC index, when a set size comparison within participants was not possible. These findings present the participants’ across-trials comparison process between different conditions as a modulator for the comparison strategy of single prime-probe sequences. The mere presence of multiple set sizes within an experiment appears to influence how participants apply decision heuristics in the form of signaling, as reflected in the PRC index. When set sizes can be directly compared, set size itself becomes a determining factor in shaping signaling strategies. The absence of PRC for the small set size suggests that signaling heuristics are no longer applied to this condition when larger set sizes are also present, eliminating associated costs and benefits. This pattern indicates that, in the presence of varying set sizes, participants adjust their criterion for employing heuristics, favoring larger set sizes where additional stimuli are required to generate a response-biasing signal. In contrast, when no direct comparison is possible in a between-subjects design, this shift does not occur, and even small set sizes—commonly used in signaling research (e.g., Hazeltine et al., 2024; Weissman, 2023)—continue to elicit signaling strategies. The experimental context can thus be introduced as an influential factor for signaling heuristics in sequential tasks.

Please note that in partial repetition tasks, not only signaling, but also stimulus-response binding (DRB) is a process contributing to performance costs and advantages (Weissman et al., 2023). This account introduces a quantitative instead of a qualitative signaling assumption, where a signaling heuristic is not regarded as the only major cause for the observed performance pattern. Rather, the authors suggest that each stimulus feature in a present display is compared to the previous and all these comparisons bias response selection towards a repetition or change. However, the extent to which binding and signaling each contribute to these effects may vary depending on the experimental setup.

For instance, the extent to which signaling contributes to partial repetition cost tasks also depends on the number of response options. Partial repetition costs, which reflect a combination of signaling and binding, tend to be smaller in tasks with four response choices compared to those with only two. In a four-choice task, a change signal does not strongly suggest a specific alternative response, whereas in a two-choice task, the benefit of a complete change is more pronounced (Hazeltine et al., 2024; Weissman et al., 2023). In our study, we deliberately employed a two-choice task to maximize the likelihood of participants applying signaling heuristics. Increasing the number of response choices to four might reduce the observed PRC index by diminishing the advantage of a complete change. Notably, signaling heuristics themselves may be subject to binding processes within event files (Hazeltine et al., 2024). Please note that a reduction in complete change benefits does not occur in other paradigms such as task switching (Koch et al., 2023). Our approach meaningfully extends recent research on signaling by demonstrating that not only the number of response choices but also the availability of contextual information can impact heuristic response strategies.

However, our study results diverge from predictions of the binding account which stands in contrast to predictions from the signaling hypothesis. Intriguingly, binding and signaling make opposite predictions here. The binding account does not predict an increase in binding effects with a larger number of different distractors, and can even lead to a decrease (Hell et al., submitted). To date, relatively little research has explored why partial repetition costs diminish as the number of stimuli increases. From a binding perspective, a distractor increase may lead to a competition for integration and retrieval of the distractors, with additional distractors introducing noise that weakens overall binding strength (Singh & Frings, 2018). As a result, the probability that all distractors are integrated into a single event file decreases when multiple distractors are present (Schmalbrock et al., 2023). Additionally, evidence suggests that event file integration is constrained by capacity limitations, meaning that an excessive number of distractors may exceed the available cognitive resources for integration (Hell et al., submitted). However, according to the signaling account, partial repetition costs should increase with a larger number of distractors because of an increased similarity and a stronger ‘response signal’.

Binding therefore does not provide explanations for the results observed in this study. Instead, our findings are in line with predictions of a signaling heuristic focusing on a direct comparison process between the current and prior display.

*What circumstances increase the contribution of signaling?*

There are several reasons for a strong dominance of signaling over binding processes in this current work. The first is the general experimental setup, which strongly supports the application of a display comparison process. From prime to probe, all distractors stayed in position and either completely repeated or changed, providing a strong signal cue, especially in larger set sizes compared to smaller ones. Additionally, there were only two possible response options, implying the specific alternative response in case of a response change. Under such circumstances, a strong ‘signaling affordance’ emerges which drastically increases the chance for an application of a simple bypass rule.

Secondly and importantly, the perceived display similarity seems to play an important role in the signaling process: We observe a smaller or eliminated PRC index for small set sizes if they are compared to larger ones within participants. Displays with many stimuli contain more information about a stimulus repetition or change and thus may have a stronger signaling potential. The results suggest that even in larger displays, a repetition or change seems to be perceived easily and holistically without the need to serially process each distractor in such a setup. This holistic display perception enables an effective use of a large amount of information, so that the (dis-)similarity between displays becomes more (or less) salient as set size increases (or decreases; for a visual comparison, see Fig. 1). Thus, the perceived similarity and signaling potential is reduced with 2 repeating stimuli in a context in which some displays also contain more stimuli.

Our findings pronounce the relevance of top-down directed processes driven by experience and episodic memory that adapt current response strategies in accordance with available context information. Please note that the context of different set sizes and increased display similarity in Exp. 1 do not result in a pure amplification of the signal in the large set size. Rather, the fact that partial repetition costs in form of the PRC index disappears completely for set size 2 in the within-subjects design also speaks for a reduction or a down-tuning in small set sizes.

In conclusion, this work investigated signaling in different contexts, with either displaying broader contextual information in terms of different set sizes (inducing similarity comparisons) or restricting information to a single set size condition. With broader context information, partial repetition costs were reduced and eliminated with small set sizes. This modulation did not occur when only information of one single set size was available. Signaling as a response selection heuristic is a complex mechanism incorporating contextual information. This enables comparison processes between different set size conditions that in turn lead to changes in the strength of signaling. Binding and retrieval as well as signaling contribute to what is measured as a PRC index – here, we showed the context sensitivity of signaling.

## Data Availability

The data, experimental program code, materials and analysis scripts for all experiments are available at the Open Science Framework: https://osf.io/zurqx/?view_only=5ed825a2ac3a492b8f6adda177c7ed20.

## Declarations

### Open practices

The necessary materials, program code, data, data analysis scripts and results are available at https://osf.io/zurqx/?view_only=5ed825a2ac3a492b8f6adda177c7ed20.
